# Unexpected Hemodynamic Instability Following Suspected Tumor Content Leakage During Laparoscopic Resection of Pheochromocytoma: A Case Report

**DOI:** 10.7759/cureus.103864

**Published:** 2026-02-18

**Authors:** Mikio Hirata, Hiroyuki Seki

**Affiliations:** 1 Anesthesiology, Kyorin University School of Medicine, Tokyo, JPN

**Keywords:** hemodynamic instability, hypertension, hypotension, laparoscopic surgery, pheochromocytoma

## Abstract

Pheochromocytoma resection is frequently associated with marked perioperative hemodynamic instability due to excessive catecholamine release. We report a case characterized by an unusual sequence of abrupt hypotension during tumor manipulation followed by sustained postoperative hypertension and tachycardia. A 51-year-old man underwent laparoscopic adrenalectomy for a large adrenal pheochromocytoma after appropriate preoperative α- and β-adrenergic blockade and volume optimization. Following pneumoperitoneum formation, severe hypertension developed despite intensified anesthetic management. During tumor handling, blood pressure suddenly declined, necessitating vasopressor support. Based on intraoperative findings, leakage of catecholamine-rich tumour contents was suspected, leading to a transient reduction in systemic catecholamine inflow. After tumor resection, hypertension and tachycardia persisted for several hours postoperatively, requiring β-blocker therapy before gradually resolving. This atypical hemodynamic pattern suggests that leaked catecholamines may have been absorbed from the peritoneal cavity, resulting in prolonged sympathetic activation. This case highlights that intraoperative tumor content leakage can cause unpredictable and paradoxical hemodynamic responses during pheochromocytoma surgery. Awareness of this mechanism and close communication between surgeons and anesthesiologists are essential for prompt recognition and safe perioperative management.

## Introduction

Pheochromocytomas are rare catecholamine-producing neuroendocrine tumors arising from chromaffin cells of the adrenal medulla or paraganglia. Excess secretion of epinephrine and norepinephrine leads to marked stimulation of α- and β-adrenergic receptors, resulting in vasoconstriction, tachycardia, and labile blood pressure. Surgical resection is the definitive treatment; however, anesthetic management is frequently complicated by pronounced hemodynamic instability [1,2]. Excessive catecholamine release may be provoked by perioperative stimuli such as laryngoscopy, tracheal intubation, pneumoperitoneum, tumor manipulation, or inadequate anesthetic depth and analgesia [3-5]. Conversely, after tumor removal, abrupt withdrawal of endogenous catecholamine secretion, combined with residual α-adrenergic blockade and relative intravascular volume depletion, may lead to severe hypotension due to reduced systemic vascular resistance. Against this physiological background, we report a case of sudden hypotension during tumor manipulation followed by sustained postoperative hypertension and tachycardia - an atypical hemodynamic sequence in pheochromocytoma surgery.

## Case presentation

**Figure 1:**
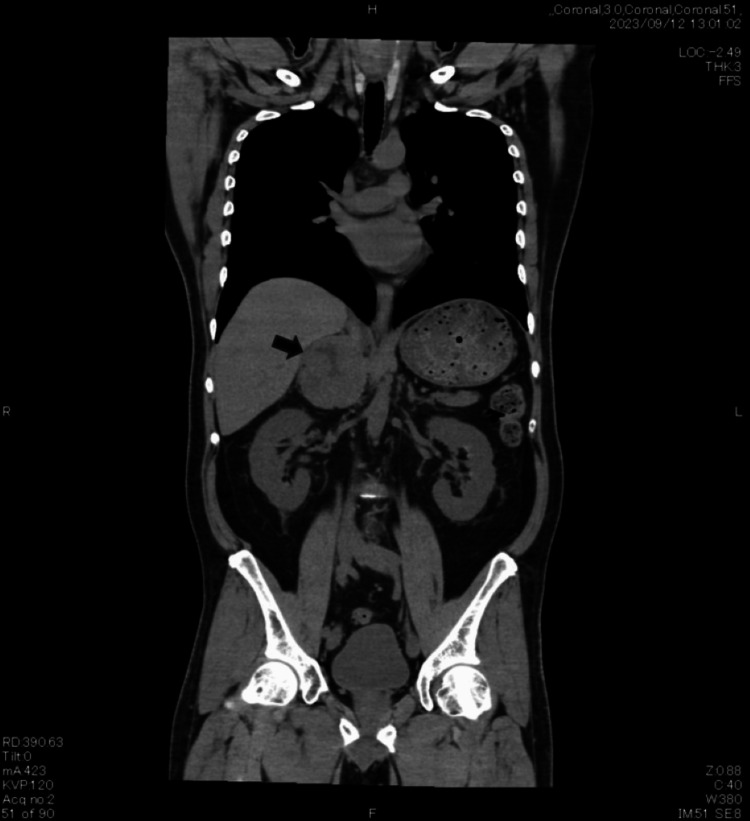
Computed tomography of the right adrenal pheochromocytoma Computed tomography shows a well-defined mass measuring approximately 7 cm in diameter in the right adrenal gland (arrow).

A 51-year-old man (height, 169 cm; weight, 78 kg) with hypertension, diabetes mellitus, and dyslipidemia presented with headaches and palpitations. Computed tomography revealed a 7-cm mass in the right adrenal gland (Figure 1).

Urinary metanephrine and normetanephrine, as well as plasma catecholamines, were markedly elevated (Table 1), confirming the diagnosis of pheochromocytoma. Accordingly, the patient was referred to our hospital for surgery.

**Table 1:** Preoperative plasma and urinary catecholamine measurements

Parameter	Measured Value	Reference range
Urinary metanephrine (24-h)	24.6 mg/day	0.04-0.19 mg/day
Urinary normetanephrine (24-h)	13.1 mg/day	0.09-0.33 mg/day
Plasma epinephrine	5,225 pg/mL	0-100 pg/mL
Plasma norepinephrine	8,032 pg/mL	100-450 pg/mL
Plasma dopamine	23 pg/mL	0-20 pg/mL

Doxazosin (16 mg/day) and nifedipine (20 mg/day) were prescribed at the previous institution. However, as blood pressure remained suboptimally controlled, nifedipine administration was increased to 40 mg/day at our hospital. Bisoprolol (1.25 mg/day) was initiated for the tachycardia. The diabetes was managed with dapagliflozin (10 mg/day) and teneligliptin (20 mg/day) (HbA1c, 7.8%). The patient was admitted one week before surgery, and intravenous saline (1,500 mL/day) was started three days preoperatively to optimize the intravascular volume.

In the operating room, standard electrocardiogram, noninvasive blood pressure, and pulse oximetry monitors were attached, and a radial arterial catheter was placed for continuous blood pressure monitoring. Vital signs on arrival in the operating room were as follows: heart rate, 95/min; blood pressure, 140/81 mmHg; and SpO₂, 98%. An epidural catheter was inserted at T11/12. General anesthesia was induced with propofol (80 mg), remifentanil (80 μg), and rocuronium (50 mg). Tracheal intubation did not cause significant hemodynamic fluctuations, and systolic blood pressure remained at approximately 100 mmHg. Anesthesia was maintained with sevoflurane and continuous remifentanil infusion. Intermittent epidural administration of 1% lidocaine provided effective analgesia throughout the procedure.

**Figure 2:**
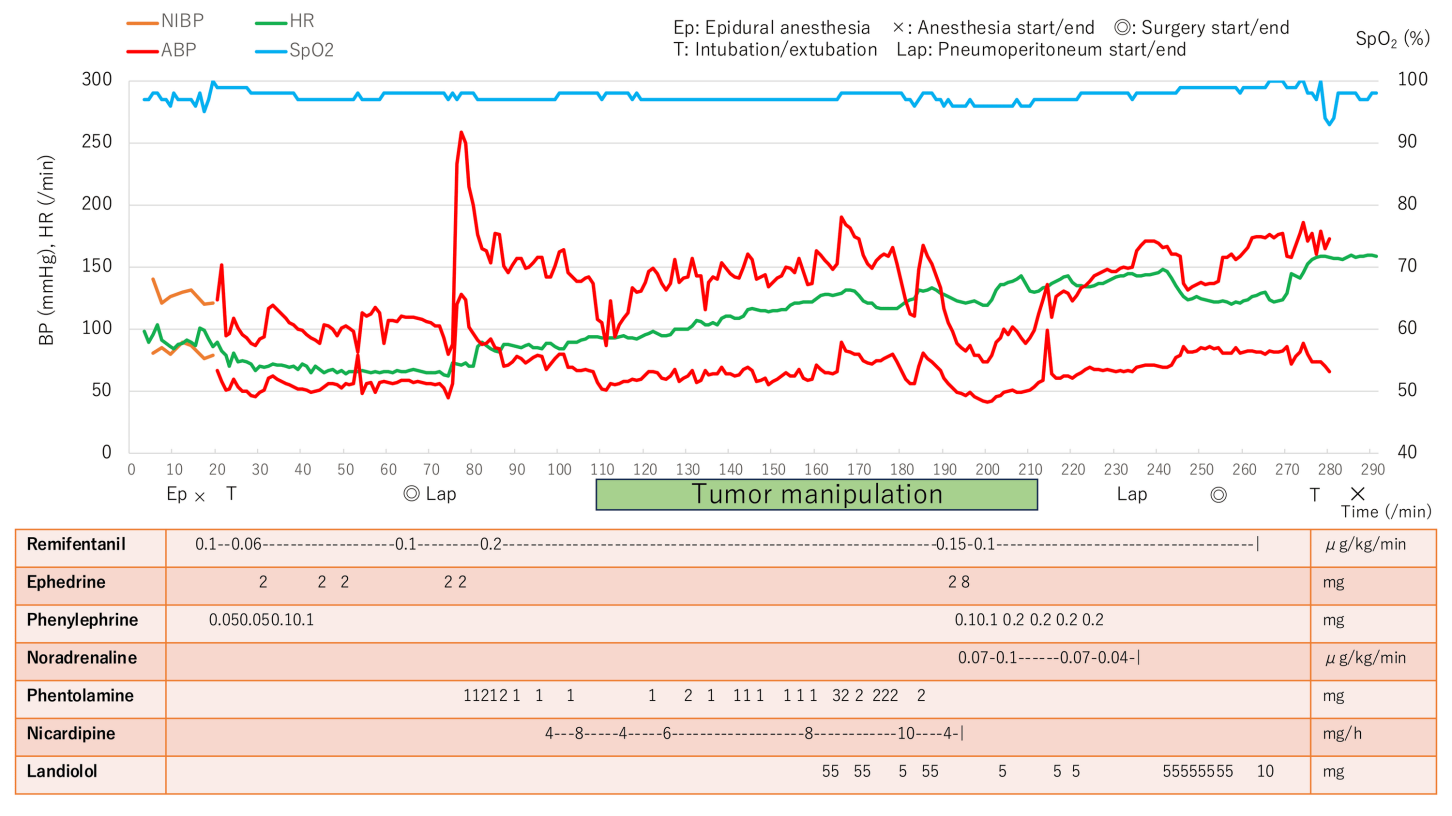
Intraoperative hemodynamic course BP: blood pressure, HR: heart rate, NIBP: non-invasive BP, ABP: arterial BP, SpO₂: oxygen saturation

A central venous catheter was inserted via the right internal jugular vein, during which the blood pressure was maintained between 90 and 100 mmHg with small boluses of phenylephrine and ephedrine. After skin incision, the blood pressure remained stable at approximately 100/50 mmHg. Immediately after pneumoperitoneum formation, the systolic blood pressure decreased to 80 mmHg, and 2 mg of ephedrine was administered. Shortly thereafter, a rapid hypertensive surge occurred, with systolic blood pressure peaking at 258 mmHg despite increased remifentanil and sevoflurane and repeated phentolamine boluses. Nicardipine infusion was added, which controlled systolic blood pressure at approximately 150 mmHg during tumor manipulation (Figure 2).

During tumor handling, blood pressure decreased suddenly and precipitously. Vasopressors (ephedrine, phenylephrine, and norepinephrine) were promptly administered, and systolic blood pressure was stabilized at a nadir of 74 mmHg. No significant intraoperative bleeding was observed in the surgical field, and there were no clinical signs suggestive of anaphylaxis, such as bronchospasm, cutaneous manifestations, or abrupt changes in airway pressure. Although the precise cause of the hypotensive episode was not immediately evident, intraoperative tumor content leakage with a transient reduction in systemic catecholamine inflow was considered a possible explanation. After initiation of continuous norepinephrine infusion (0.1 μg/kg/min), further hypotension did not occur; however, blood pressure and heart rate subsequently increased. Hypertension persisted after tumor resection despite discontinuation of norepinephrine.

At the end of the surgery, the blood pressure remained at 170/70 mmHg and the heart rate was approximately 150/min. After the trachea was extubated in the operating room, the patient complained of dyspnea; however, chest radiography revealed no pulmonary edema or atelectasis, and oxygenation was adequate (SpO₂ 98% on room air). The patient was subsequently transferred to the intensive care unit.

In the intensive care unit, the blood pressure remained at approximately 180/90 mmHg and the heart rate was 100-140/min, necessitating landiolol administration (2 μg/kg/min). Patient hemodynamics improved gradually after approximately three hours then stabilized after 11 hours, allowing the discontinuation of landiolol at 20 hours. The patient was transferred to the general ward after 24 hours then discharged on postoperative day 8 without complications.

## Discussion

The patient exhibited an atypical hemodynamic pattern characterized by sudden hypotension during tumor manipulation followed by sustained hypertension after tumor removal. Pheochromocytoma is associated with marked perioperative hemodynamic lability due to excessive secretion of epinephrine and norepinephrine, which stimulate α-adrenergic-mediated vasoconstriction and β-adrenergic chronotropic and inotropic effects [1,2]. Intraoperative hypertension is typically triggered by catecholamine surges induced by tumor manipulation or pneumoperitoneum [3-5], whereas post-resection hypotension is generally attributed to abrupt withdrawal of endogenous catecholamine release combined with residual α-blockade and relative intravascular volume depletion.

Predicting intraoperative hemodynamic instability (IHI) remains challenging. Tumor size, preoperative catecholamine levels, adequacy and duration of α-blockade, and cardiovascular comorbidities have been associated with IHI; however, findings are inconsistent and no predictor has been universally validated [6-9]. Although the present patient had a large tumor and markedly elevated catecholamine concentrations, these characteristics alone do not reliably determine the intraoperative hemodynamic course.

Sudden hypotension during tumor manipulation was unexpected. No significant surgical bleeding or clinical features suggestive of anaphylaxis were observed, making these common causes of intraoperative hypotension unlikely. Based on operative findings suggesting disruption of tumor integrity, intraoperative tumor content leakage was considered a plausible explanation. Similar hemodynamic alterations have been reported when catecholamine-rich cystic fluid was aspirated during resection of a giant pheochromocytoma, resulting in abrupt hypotension [10]. A possible mechanism is acute depletion of the intratumoral catecholamine reservoir, leading to a transient reduction in systemic vascular resistance that had been sustained by continuous catecholamine release.

We did not measure intraoperative catecholamine concentrations; therefore, a direct biochemical link between tumor content leakage and the observed hemodynamic changes cannot be confirmed. Nevertheless, in the absence of bleeding or anaphylaxis and in light of prior reports, tumor content leakage remains a reasonable explanatory hypothesis.

Following tumor removal, the patient developed sustained hypertension and tachycardia, which contrasts with the more typicalpattern of post-resection hypotension described in the literature [3-5]. Adequate epidural analgesia was maintained, making pain-induced sympathetic activation unlikely. There was no evidence of pulmonary edema or catecholamine-induced cardiomyopathy [11]. One possible explanation is delayed systemic absorption of catecholamines that had leaked into the peritoneal cavity, resulting in prolonged sympathetic stimulation. Although this mechanism remains inferential, it is physiologically consistent with the pharmacodynamic effects of circulating catecholamines.

Preoperative optimization with α-blockade and volume expansion is considered essential to reduce perioperative risk [1,2]. In the present case, appropriate preoperative medical preparation was undertaken; however, this case illustrates that even adequately optimized patients may experience unpredictable and rapidly shifting hemodynamic patterns. Prompt pharmacologic intervention with vasopressors during hypotension and vasodilators and β-blockade during hypertension, combined with continuous invasive monitoring, was critical to maintaining stability.

Laparoscopic adrenalectomy is widely accepted as the standard surgical approach [3-5]. However, pneumoperitoneum and tumor manipulation may increase intratumoral pressure, potentially predisposing to minor capsular disruption or leakage. In addition to hemodynamic consequences, capsular rupture has been associated with peritoneal implantation of pheochromocytoma cells [12], emphasizing the importance of meticulous surgical handling and interdisciplinary communication.

Most reports of intraoperative tumor rupture in pheochromocytoma have primarily focused on oncologic outcomes, particularly the risk of peritoneal dissemination and recurrence following capsular disruption [12-14]. Detailed descriptions of accompanying intraoperative hemodynamic alterations have been limited or absent in those reports. In contrast, the present case provides a detailed temporal account of abrupt hypotension during tumor manipulation followed by sustained postoperative hypertension, thereby highlighting the potential hemodynamic consequences of tumor rupture or content leakage.

Although causality cannot be definitively established in the absence of intraoperative catecholamine measurements, the combination of operative findings, exclusion of alternative causes such as bleeding or anaphylaxis, and the temporal relationship between tumor manipulation and hemodynamic collapse supports the plausibility of this mechanism. By focusing specifically on the perioperative hemodynamic sequence rather than oncologic outcomes alone, this report expands the current understanding of the clinical implications of tumor disruption during pheochromocytoma surgery.

Taken together, this case expands the spectrum of reported intraoperative hemodynamic patterns in pheochromocytoma surgery. When abrupt hypotension occurs during tumor manipulation without evidence of bleeding or anaphylaxis, tumor content leakage should be considered among the differential mechanisms. Recognition of this possibility may assist anesthesiologists and surgeons in anticipating and managing complex perioperative hemodynamic instability.

## Conclusions

In conclusion, pheochromocytoma surgery may be complicated by unpredictable hemodynamic fluctuations when the tumor contents leak into the peritoneal cavity. Leakage may cause paradoxical hypotension during tumor manipulation, followed by sustained hypertension after tumor removal. Close interdisciplinary communication is essential for safely anticipating and managing these events.
